# Efficiency evaluation of 28 health systems by MCDA and DEA

**DOI:** 10.1186/s13561-024-00538-y

**Published:** 2024-07-29

**Authors:** Martin Dlouhý, Pavel Havlík

**Affiliations:** 1 Faculty of Informatics and Statistics, Prague University of Economics and Business, 4 Winston Churchill Sq, Prague, Czech Republic; 2 Faculty of Finance and Accounting, Prague University of Economics and Business, 4 Winston Churchill Sq, Prague, Czech Republic

**Keywords:** Health system efficiency, Data envelopment analysis, Multiple-criteria decision analysis, OECD

## Abstract

**Background:**

Policymakers, who are constantly discussing growing health expenditures, should know whether the health system is efficient. We can provide them with such information through international health system efficiency evaluations. The main objectives of this study are: (a) to evaluate the efficiency of health systems in 28 developed countries by multiple-criteria decision analysis (MCDA) and data envelopment analysis (DEA) and (b) to identify reasonable benchmark countries for the Czech Republic, for which we collect information on the relative importance of health system inputs and outputs.

**Methods:**

We used MCDA and DEA to evaluate the efficiency of the health systems of 28 developed countries. The models included four health system inputs (health expenditure as a relative share of GDP, the number of physicians, nurses, and hospital beds) and three health system outputs (life expectancy at birth, healthy life expectancy, and infant mortality rate). The sample covers 27 OECD countries and Russia, which is also included in the OECD database. To determine the input and output weights, we used a questionnaire sent to health policy experts in the Czech Republic.

**Results:**

We obtained subjective information on the relative importance of the health system inputs and outputs from 27 Czech health policy experts. We evaluated health system efficiency using four MCDA and two DEA models. According to the MCDA models, Turkey, Poland, and Israel were found to have efficient health systems. The Czech Republic ranked 16th, 19th, 15th, and 17th. The benchmark countries for the Czech Republic’s health system were Israel, Estonia, Luxembourg, Italy, the UK, Spain, Slovenia, and Canada. The DEA model with the constant returns to scale identified four technically efficient health systems: Turkey, the UK, Canada, and Sweden. The Czech Republic was found to be one of the worst-performing health systems. The DEA model with the variable returns to scale identified 15 technically efficient health systems. We found that efficiency results are quite robust. With two exceptions, the Spearman rank correlations between each pair of models were statistically significant at the 0.05 level.

**Conclusions:**

During the model formulation, we investigated the pitfalls of efficiency measurement in health care and used several practical solutions. We consider MCDA and DEA, above all, as exploratory methods, not methods providing definitive answers.

## Introduction

Health systems face several challenges due to the increasing costs of health care, the aging of the population associated with a rise in chronic diseases, and unequal access to health care due to the uneven regional distribution of health professionals and infrastructure. Each country is under pressure to provide quality health care while containing health expenditures. For this reason, it is necessary to study the health system efficiency to investigate whether money and other health system inputs are allocated efficiently and not wasted. A good analysis can support more effective health policy decisions.

Health systems consume increasing amounts of money worldwide. In 2022, health expenditures composed 9.2% of the GDP in the OECD member countries [1]. These funds are provided primarily from public sources. The average share of public health expenditures in the OECD countries is above 70%. Nevertheless, not all countries obtain good value for money. Among the OECD countries, the highest health expenditures in 2022 were in the United States at 16.60% of the GDP (12,555 USD in PPP per capita), Germany at 12.70% of the GDP (8,011 USD per capita), and France at 12.01% of the GDP (6,630 USD per capita). However, some countries achieved similar or even better health outcomes with much lower costs. For example, Korea spent 9.70% of its GDP (4,570 USD in PPP per capita), Italy spent 9.00% of its GDP (4,291 USD per capita), and Israel spent 7.40% of its GDP (3,444 USD per capita). The health expenditures in these three countries were lower than those in the United States, Germany, and France; however, Korea, Italy, and Israel had the life expectancy at birth of around 83 years in 2022, while the life expectancy was 76.4 years in the United States, 80.8 years in Germany, and 82.4 years in France [1]. This comparison raises questions about why health systems in some countries perform better than in others.

Of course, there are other health determinants other than health care, such as lifestyle, social and economic development, and environment. Moreover, health systems across countries differ in their history, norms, market regulation, and financing mechanisms. As stated by Schneider et al. [2], no two countries are alike when it comes to health care because each country has settled on a unique mix of policies, service delivery systems, and financing models. Despite many apparent differences, health systems have identical goals, such as better health, high-quality health care, equity, and financial stability. International comparisons of health systems are important for policymakers to know how the national health system is performing, identify good and bad processes and find the right approach to a sustainable and high-quality health system [3–5].

The main objectives of this study are: (a) to evaluate the efficiency of health systems in developed countries by multiple-criteria decision analysis (MCDA) and data envelopment analysis (DEA); and (b) to identify reasonable benchmark countries for the Czech Republic, for which we collect information on the relative importance of health system inputs and outputs.

The rest of the paper is organized as follows: The *Literature review* is devoted to the review of health system efficiency evaluation studies. The Methods section describes the methods used, the choice of the sample, the selection of health system inputs and outputs, and the questionnaire survey among health policy experts. The Results section presents the results of the questionnaire survey on the relative importance of health system inputs and outputs and the results of health system efficiency evaluations obtained by MCDA and DEA. Based on the efficiency results and the amounts of the inputs and outputs, benchmark countries for the Czech health system are defined. In the *Discussion*, we describe the most common pitfalls in health system evaluation and how we address them. The Conclusion section summarizes the results and concludes the paper.

## Literature review

The purpose of health systems is to improve the health of the population they serve, respond to people’s expectations, and provide financial protection against the costs of ill health [5]. A health system that cannot meet the objectives mentioned above cannot fully contribute to the health of a society. The health systems in the OECD member countries are financed primarily from public resources, which are limited; nevertheless, there is constant pressure from society to achieve the best possible health. Policymakers thus face a difficult and conflicting task of adopting policies that enhance the quality of health services on one side and contain costs on the other.

An international comparison of health systems is a popular tool of efficiency evaluation that offers an international benchmarking of whether resources are used efficiently. Dlouhý [6] states three assumptions of international comparisons: (a) the production processes of health systems are comparable; (b) we are able to say that the performance of one health system is, at least in some aspects, better than the performance of another health system; (c) the experience obtained from performance evaluation is transferable from one health system to another.

There is a wide range of methods for evaluating health systems. They may include qualitative comparisons, quantitative methods, and combinations of both approaches. Several international organizations are concerned with international comparisons, including the World Health Organization (WHO), the European Union (EU) and the OECD (e.g. [5, 7–9]). Over the years, several methods and frameworks have been created, according to which health system assessments are given various names, such as health system profiles, health sector situational analysis, health system monitoring, health system analysis or health system performance assessment. Papanicolas et al. [9] provided a detailed description of these methods.

Quantitative efficiency evaluation studies use a composite index or set of indicators. For example, Tchouaket et al. [10] used a set of indicators to obtain homogenous groups of countries whose health systems achieved similar performance profiles. However, most efficiency evaluation studies use quantitative methods, such as multiple-criteria decision analysis (MCDA), data envelopment analysis (DEA), free disposable hull (FDH), stochastic frontier analysis (SFA) and other regression methods. The indicators used in such studies are often based on data from international organizations such as the WHO, OECD, World Bank, and European Union.

The *World Health Report 2000* [5] was the major attempt at an international comparison of health system performance at the global level. The *World Health Report 2000* identified five goals of health systems: the overall level of health (with a weight of 0.25), the distribution of health in the population (0.25), the overall level of responsiveness of the health system (0.125) and the distribution of responsiveness (0.125), and fair financial contribution (0.25). The weights of individual goals were determined by a survey among 1006 experts from 125 countries, half of whom were WHO staff. The efficient frontier was estimated by the stochastic frontier analysis [11].

A well-known health system assessment of European health systems is the *Euro Health Consumer Index* (EHCI), published by the private company Health Consumer Powerhouse [12] since 2005. EHCI evaluates health systems in European countries from the patients’ point of view. The 2018 health assessment evaluated 35 European health systems by the set of 46 indicators divided into six sub-disciplines with the following weights: 10 indicators of patient rights, information, and e-health with a total weight of 0.125, 6 indicators of accessibility (0.225), 9 indicators of health outcomes (0.300), 8 indicators of range and reach of services (0.125), 7 indicators of prevention (0.125), and 6 indicators of pharmaceutical sub-discipline (0.100). The determination of weights is not clearly described in the EHCI; it is just mentioned that weights are based on discussions with expert panels and experience from several patient survey studies. According to the 2018 edition of the EHCI, the three best health systems from the patients’ perspective are in Switzerland, the Netherlands and Norway [12]. A rough estimate of the health system efficiency is calculated by dividing the EHCI by the square root of health expenditure per capita in PPP dollars.

Romaniuk et al. [13] developed the health system synthetic outcome measure (SOM), which is based on 41 indicators, measuring the epidemiological situation, health behaviors, and factors related to the health system. They analyzed health systems in 21 countries of Central and Eastern Europe during the transformation period of 1988–2012. The weights attributed to each indicator were chosen arbitrarily, based on the significance of a given indicator and the credibility of the data. The study identified a group of countries with the highest level of SOM (the Czech Republic, Slovenia, Estonia, Hungary, and Poland) and a group of countries with the lowest value of SOM (Moldova, Armenia, Albania, Russia, Georgia, and Ukraine).

Yiğit [14] analyzed the health system efficiency of 35 OECD countries. The efficiency score was composed of two health system inputs (health expenditure as a share of GDP and health expenditure in USD purchasing power parity) and four health system outputs (life expectancy at birth, infant mortality rate, potential years of life lost per 100 000 females, potential years of life lost per 100 000 males). TOPSIS, which is a popular MCDA method, was applied with equal weights for each input and each output. Slovenia, Korea, and Israel had highest efficiency scores, while the United States, Mexico, and Turkey had the lowest efficiency scores.

Pereira et al. [15] applied an MCDA approach to rank nine European health systems with Beveridgian financing to determine the shortcomings of the Portuguese health system. First, the panel of decision-makers used the design of a cognitive map to identify eleven fundamental points of view: care appropriateness, prevention, safety, availability, timeliness, freedom, participation, access, absence of asymmetries, expenditure, and payments. Second, points of view were made operational for evaluation by selecting acceptable descriptors of performance. Third, an MCDA procedure was proposed to evaluate nine health systems using the elementary additive value model.

Retzlaff-Roberts, Chang, and Rubin [16] evaluated health system efficiency in 27 OECD countries in 1998. They calculated the input-oriented and output-oriented variable returns to scale (VRS) DEA models with four health system inputs (beds, physicians, MRI, and health expenditures), three social environment inputs (school expectancy, Gini index, and tobacco use) and one health system output (infant mortality or life expectancy). Spinks and Hollingworth [17] used and compared the OECD and WHO datasets to evaluate the efficiency of health systems in 28 OECD countries for the years 1995 and 2000. The single health system output was life expectancy in the OECD model and disability-adjusted life expectancy in the WHO model. The health system inputs in both models were GPD per capita, education, unemployment, and health expenditure per capita.

Asandului et al. [18] used DEA to assess the health system efficiency in 30 European countries in 2010. The data came from the Eurostat database. They calculated CRS and VRS DEA models with three health outputs (life expectancy at birth, HALE - health adjusted life expectancy, and infant mortality rate) and three health system inputs (number of doctors, number of hospital beds, and public health expenditures as percentage of GDP). Cetin and Bahce [19] applied DEA to assess the health system efficiency of 34 OECD member countries in 2011. The number of doctors, number of beds, and health expenditure per capita were used as health system inputs and life expectancy at birth and infant mortality rate were used as outputs. At the second stage of their analysis, eight countries were removed from the set as outliers. Behr and Theune [20] studied the health system efficiency of 34 OECD member countries. Instead of analyzing the health system as a whole, they conducted five separate partial DEA analyses: the efficiency of surgery provision, the efficiency of mortality prevention, the effects of lifestyle on life expectancy at birth, the effects of income and health expenditure per capita on life expectancy at birth, and on the effects of relative health expenditure and inequality on life expectancy at birth. The analysis by the input-oriented CRS model showed large within-country variability among the efficiencies of five aspects of the health system. The ranking of countries is based on the mean of five efficiency scores. The most efficient country is Iceland, followed by Turkey and Estonia. Cylus et al. [21] considered DEA as a tool for constructing composite health system efficiency indicators from several partial efficiency measures. They tested the idea on a set of 11 OECD member countries.

Ahmed, Hasan, MacLennan et al. [22] applied an output-oriented DEA to estimate the technical efficiency of 46 Asian health systems. They used health expenditure per capita as a single health system input and healthy life expectancy at birth (HALE) and infant mortality rate as health system outputs. In the next step, the Tobit regression was used to identify the factors associated with the health system efficiency. Gavurova et al. [23] compared the health system efficiency of 36 OECD member countries in the years 2000, 2008, and 2016. They applied the input-oriented dynamic network DEA model to the health system, which was divided into the public health sub-division and the medical care sub-division. Dlouhý [6] investigated the technical efficiency of 38 health systems in OECD member countries in 2019. In the first model, the outputs were doctor consultations and inpatient care discharges. In the second model, the output was life expectancy at birth. In both models, the health system inputs were physicians, nurses, and beds. Dlouhý describes 14 recommendations on how to deal with the non-homogeneity of health systems in DEA models.

Pereira et al. [24] used a network DEA model to evaluate the health system efficiency in the fight against COVID-19. The sample included 55 countries (37 OECD member countries, six prospective OECD members, four OECD key partners, and eight other countries). Lupu and Tiganasu [25] analyzed the health system efficiency of 31 European countries in treating COVID-19. In the first step, the DEA models evaluated three stages of the pandemic: the first wave (January 1–June 15), the relaxation period (June 15–October 1) and the second wave (October 1–December 31). In the second step, the Tobit regression was used to determine the key factors of health system efficiency. Ersoy and Aktaş [26] measured the health system efficiency of 37 OECD countries for 2020 using the input-oriented super-efficiency CRS and VRS DEA models with four health system inputs (doctors, nurses, beds, current health expenditure as a share of GDP) and three outputs (infant mortality rate, mortality rate under 5 years, life expectancy at birth). During the first year of the COVID-19 pandemic, 14 countries in the CSR DEA model and 20 countries in the VRS DEA model were efficient. Selamzade et al. [27] measured the efficiency of health system in 38 OECD countries in the fight against the COVID-19 pandemic in 2021. They applied the output-oriented super-efficiency CRS DEA model and three MCDA methods (TOPSIS, EDAS, and CODAS). The models included three inputs (doctors, nurses, beds) and four outputs (health expenditure per capita in USD, COVID-19 tests, cases, and deaths). Colombia, Denmark, New Zealand, Slovakia, and the USA were efficient; on the other hand, Hungary and Chile were the health systems with the lowest efficiency.

GBD 2015 Healthcare Access and Quality Collaborators [28] used FDH with bootstrapping to produce an efficiency frontier based on the relationship between the *Healthcare Access and Quality Index* (HAQ) Index and the *Socio-demographic Index* (SDI). The HAQ index is measured on a scale from 0 (worst) to 100 (best) and uses 32 causes of amenable mortality that could be avoided by timely and effective health care. The SDI is a measure of overall development consisting of income per capita, average years of education, and total fertility rates. Pereira and Camanho [29] revisited the computation of the HAQ index by a fuzzy data envelopment analysis model and proposed the efficiency HAQ index (E-HAQI). The single input was total health expenditure per capita.

In summary, the non-parametric DEA dominates the literature on health system efficiency evaluation. Mbau et al. [30] reviewed 131 studies from 2000 to 2021 dealing with efficiency assessment in the health system at the national or regional level. Quantitative methods were used in 94% of the studies, only 4% of the studies used qualitative methods, and 2% of studies used mixed methods. DEA was used exclusively in 95 papers (76%), and in 2% of the papers, DEA was applied in combination with FDH and SFA. Stochastic frontier analysis was used exclusively in 23 studies (18%). The application of MCDA is not mentioned. Varabyova and Müller [31] reviewed 22 efficiency studies at the national level, 13 of which applied non-parametric methods (DEA and FDH), eight of which applied parametric methods (SFA or other regression methods), and one study used both parametric and non-parametric methods. No study in their review used MCDA.

## Methods

### Selection of countries

The best sources of comparative data on health systems in developed countries are the databases of the World Bank, OECD, Eurostat, and the World Health Organization. All these databases also include data on the Czech Republic. We decided to use the OECD database, which includes data on 38 OECD member countries, accession candidate countries, and other partner countries. The OECD database ensures good data accuracy and comparability, which is essential for the correct efficiency evaluation at the international level. Our sample covers 27 OECD countries and Russia, which is also included in the database. The rest of the OECD member countries and the non-OECD countries included in the OECD database were omitted due to missing 2019 data at the time of analysis. We deliberately chose 2019 because it was the last year that was not affected by the COVID-19 pandemic.

### Selection of health system indicators

For the efficiency evaluation, we have chosen four health system inputs that are common in studies on health efficiency assessment [20, 30]. The selected health system inputs are health expenditure, the number of physicians, the number of nurses, and the number of hospital beds. Since health expenditure is expressed as a relative share of GDP, we avoid the problem of expressing health expenditure in US dollars. It is no surprise that it is a very popular indicator in international comparisons. The number of physicians is another frequently used health system input. The indicator represents people with a university education in the medical specialization and authorization to perform practice. The indicator is measured as the number of physicians per 1,000 inhabitants. A third health system input is the number of nurses. The OECD defines nurses as all practicing nurses providing direct health services to patients, including self-employed nurses [1]. This health system input is important because some activities that are usually carried out by physicians in one country are carried out by nurses in another country. Using only the number of physicians may be problematic in terms of comparability across countries. Moreover, as shown below, the experts attributed almost the same importance (weight) to nurses as to physicians. The last health system input is the number of hospital beds.

The model uses three health system outputs: life expectancy at birth, healthy life expectancy, and infant mortality rate. Life expectancy at birth is considered one of the key indicators of a country’s health system. Healthy life expectancy (HALE) is an appropriate indicator for monitoring health as the production factor. An increase in this indicator leads to decreased healthcare costs and increased human productivity. Our survey shows that HALE is the most important health system output without ambiguity. This indicator came from the WHO database. The last health system output is the infant mortality rate. This indicator can be interpreted as the probability of a child born in a specific year dying before reaching the age of one.

In many applications, including this paper, it is desirable to incorporate ratio measures with managerial or policy meaning, even though ratio measures generally do not satisfy the standard production assumptions [32]. The aim of this study is primarily to identify peer health systems for benchmarking purposes. However, the health system represents a relatively large and complex system, so we cannot directly interpret the efficiency score of 0.50 that health system efficiency is achieved if inputs are halved, or outputs are doubled. The practical interpretation of the results is looser in contrast to strict theoretical assumptions. This weakness of complex systems in interpretation limits, on the other hand, the risks associated with using ratio measures. This is consistent with our view that DEA and MCDA are, above all, exploratory efficiency measurement methods, not methods providing definitive answers.

### MCDA and weight determination

Multiple-criteria decision analysis evaluates alternatives characterized by multiple conflicting criteria. In this study, the alternatives are health systems, and the criteria are health system inputs and outputs. The simple and best-known method of MCDA is the weighted sum method, which we will use in this study. The critical issue of the weighted sum method is to determine the weights of the criteria. The weights are assigned according to the relative importance of the criteria, allowing us to sum the multiple criteria into one index. Unlike data-driven methods (DEA and SFA), MCDA actively seeks to make explicit and manage subjective value judgments rather than eliminate them [33].

First, because the infant mortality rate is a minimization criterion that breaks the isotonicity condition, we transformed the infant mortality rate (IMR) into the infant survival rate (ISR):

1 $$\>IS{R_j} = 1 - IM{R_j},\>\>j = {\rm{1,2}}, \ldots ,\>n.$$

Second, the health system inputs and outputs values *x*_*ij*_ and *y*_*kj*_ are normalized to the 0–1 range by dividing all the values by the maximum:

2 $$\eqalign{& x_{ij}^* = {{{x_{ij}}} \over {ma{x_j}\left( {{x_{ij}}} \right)}}, i = 1,2,3,4; \cr & \,\,\,\,\,\,\,\,y_{kj}^* = {{{y_{kj}}} \over {ma{x_j}\left( {{y_{kj}}} \right)}}, k = 1,2,3. \cr}$$

Third, the efficiency of a health system is the ratio between the aggregated health system output and the aggregated health system inputs. Hence, for the evaluation of the health system efficiency, we use the MCDA model in the ratio form (3), where *θ*_*j*_ is the efficiency score of the health system *j*, $\:{x}_{ij}^{*}$ is the normalized value of health system input *i* used by health system *j*, $\:{y}_{kj}^{*}$ is the normalized value of health system output *k*, and *v*_*ij*_ and *w*_*kj*_ are the input and output weights, respectively:

3 $$\>\theta {\>_j} = {{\sum \> _{k = 1}^3{w_{kj}}\>y_{kj}^*} \over {\sum \> _{i = 1}^4{v_{ij}}x_{ij}^*}}\>\>\>\>\>\>\>\>\>\>\>\>\>\>\>\>\>j = {\rm{1,2}}, \ldots ,\>n.$$

Fourth, the key issue of the MCDA is the determination of the input and output weights. For this purpose, we developed a questionnaire that was sent to experts in health policy in the Czech Republic. We sent the questionnaire to 83 experts in health policy that we can divide into three professional groups: 22 academicians, 23 health managers or public sector officials (e.g., directors of university hospitals, directors of health insurance funds, president of the Czech Medical Association), and 38 politicians (the minister of health, deputy ministers of health, members of the Committee on Health Care of the Chamber of Deputies of the Parliament of the Czech Republic, members of the Committee on Health of the Senate of the Parliament of the Czech Republic). Each respondent allocated 100 points among four health system inputs and 100 points among three health system inputs. The input and output weights *v*_*ij*_ and *w*_*kj*_ are determined by the average number of points divided by 100.

### Selection of the DEA model

Data envelopment analysis is a method of technical efficiency evaluation that is based on linear programming. DEA identifies the production possibilities frontier on which efficient production units are located [34]. The more distant the production units are from the frontier, the less technically efficient they are. DEA offers the ability to assess units with multiple inputs and outputs, does not require a parametric production function, and defines efficient benchmarks for inefficient units. According to the characteristics of the returns to scale, we can distinguish between the constant returns to scale (CRS) DEA model [35] and the variable returns to scale (VRS) DEA model [36]. We applied both DEA models. Unlike DEA, the free disposable hull (FDH) model assumes that the evaluation of a unit is based only on real units, not on their convex combinations [37]. As a result, FDH does not require any prior assumption about the returns to scale, and the production possibilities frontier is non-convex.

Let us assume that we have a set of *n* production units (health systems) that use *m* types of health system inputs to produce *r* types of health system outputs. The envelopment formulation of the output-oriented CRS DEA model (4) for health system *q* is as follows:

4 $$\eqalign{& maximize\theta {\>_q} \cr & subject\>to \cr & \sum \> _{j = 1}^n{x_{ij}}\lambda {\>_j} \le \>{x_{iq}},\>\>\>\>\>\>\>\>\>\>\>\>\>\>\>\>\>\>i = {\rm{1,2}},n,\>m, \cr & \sum \> _{j = 1}^n{y_{kj}}\lambda {\>_j} \ge \>\theta {\>_q}{y_{kq}},\>\>\>\>\>\>\>\>\>\>\>\>k = {\rm{1,2}}, \ldots \>,r, \cr & {\lambda _j} \ge \>0,\>\>\>\>\>\>\>\>\>\>\>\>\>\>\>\>\>\>\>\>\>\>\>\>\>\>\>\>\>\>\>\>\>j = {\rm{1,2}}, \ldots ,\>n, \cr}$$

where *θ*_*q*_ is the technical efficiency score, *x*_*ij*_ is the quantity of input *i* used by health system *j*, *y*_*kj*_ is the quantity of output *k* produced by health system *j*, a positive value of the variable *λ*_*j*_ identifies health systems serving as efficient peers for health system *q*. The efficiency score *θ*_*q*_ measures the size of the output expansion that makes health system *q* technically efficient; hence, the efficiency score is equal to or greater than one. However, we usually express efficiency in the form of 1/*θ*_*q*_ to take values in the 0–1 interval.

DEA is frequently used to assess efficiency in the public sector, in which outputs are typically not in monetary units. DEA is one of the most frequently used non-parametric methods for assessing the efficiency of health systems [30, 38].

DEA distinguishes the output-oriented model, which maximizes outputs produced by the constant levels of inputs, and the input-oriented model, which minimizes inputs required to produce the constant levels of outputs. The input-oriented model is considered as inappropriate as the primary goal of the health system is to maximize health, not hold health constant and minimize inputs [17]. Hence, we applied the output-oriented model that maximizes health outputs with a budget constraint, represented here by the fixed level of health system inputs.

## Results

Table 1 shows the data used in this study, which came from the OECD and WHO databases for the year 2019. The highest relative health expenditure as a share of GDP is in the United States (16.76%) and the lowest in Turkey (4.34%). Korea has the highest relative number of hospital beds (12.44 per 1,000 inhabitants), and Sweden has the lowest (2.09 per 1,000 inhabitants). The relative number of nurses is the highest in Switzerland (17.96 per 1,000 inhabitants) and the lowest in Turkey (2.4 per 1,000 inhabitants). The number of physicians is the highest in Austria (5.32 per 1,000 inhabitants) and the lowest in Turkey (1.95 per 1,000 inhabitants). The life expectancy is the highest in Switzerland (84.0 years) and the lowest in Russia (73.2 years). The healthy life expectancy is the highest in Korea (73.1 years) and the lowest in Russia (64.2 years). The last output is the infant survival rate, which is the highest in Iceland (99.89%) and the lowest in Turkey (99.1%). In the calculations, we normalized the health system inputs and outputs values to the 0–1 range by dividing all the values by the maximum.

**Table 1:** Health system inputs and outputs, 2019

Country	Expenditure as % of GDP	Beds per 1,000	Nurses per 1,000	Physicians per 1,000	Life Expectancy at birth	HALE	Infant Survival Rate
Australia	9.41	3.75	12.22	3.83	83.0	70.9	99.67
Austria	10.43	7.19	10.37	5.32	82.0	70.9	99.71
Belgium	10.65	5.57	11.10	3.16	82.1	70.6	99.63
Canada	10.84	2.52	9.98	2.78	82.1	71.3	99.56
Czech Republic	7.83	6.58	8.56	4.07	79.3	68.8	99.74
Denmark	9.95	2.59	10.15	4.27	81.5	71.0	99.70
Estonia	6.73	4.53	6.24	3.47	78.8	69.2	99.84
France	11.11	5.84	11.07	3.36	82.9	72.1	99.62
Germany	11.69	7.91	13.95	4.39	81.4	70.9	99.68
Hungary	6.35	6.91	6.62	3.49	76.4	67.2	99.64
Iceland	8.56	2.80	15.36	3.89	83.2	72.0	99.89
Israel	7.46	2.96	5.01	3.29	82.9	72.4	99.69
Italy	8.66	3.16	6.68	4.05	83.6	71.9	99.76
Korea	8.16	12.44	7.94	2.46	83.3	73.1	99.73
Latvia	6.69	5.42	4.39	3.27	75.5	66.2	99.66
Lithuania	7.00	6.35	7.74	4.57	76.4	66.7	99.67
Luxembourg	5.37	4.26	11.90	3.10	82.7	71.6	99.53
Norway	10.52	3.47	17.88	4.97	83.0	71.4	99.80
Poland	6.46	6.17	5.18	2.42	78.0	68.7	99.62
Russia	5.60	8.00	8.48	4.16	73.2	64.2	99.51
Slovakia	6.95	5.76	5.74	3.57	77.8	68.5	99.49
Slovenia	8.52	4.43	10.28	3.26	81.6	70.7	99.79
Spain	9.13	2.95	5.89	4.40	83.9	72.1	99.74
Sweden	10.92	2.07	10.90	4.38	83.2	71.9	99.79
Switzerland	11.29	4.59	17.96	4.35	84.0	72.5	99.67
Turkey	4.34	2.88	2.40	1.95	78.6	68.4	99.10
UK	10.15	2.45	8.20	2.95	81.4	70.1	99.63
USA	16.76	2.82	11.98	2.64	78.9	66.1	99.43

The questionnaire was sent to 83 Czech health policy experts in May 2023 [39]. Out of the 83 experts, 27 replied (32.53%). Out of the 23 health managers or public sector officials approached, 12 responded (52.2%). Out of the 22 academicians, 10 responded, representing 45.5%. Politicians had the lowest response rate because only five responded (13.2%). We asked the respondents to estimate the relative weights of four health system inputs: health expenditure as a percentage of GDP, the number of physicians per 1,000 inhabitants, the number of nurses per 1,000 inhabitants and the number of hospital beds per 1,000 inhabitants. The respondents distributed 100 points among four health system inputs and identified health expenditure as a share of GDP as the most important health system input, with a relative weight of 41.6%. The second most important input was the number of physicians per 1,000 inhabitants, with a weight of 22.5%. Physicians were closely followed by nurses, with a weight of 21.4%. Hence, health policy experts consider nurses to be almost as important as physicians. The number of hospital beds per 1,000 inhabitants, with a weight of 14.5%, was identified as the least important of the four evaluated inputs. Some respondents allocated no points to one or two health system outputs (Table 2). Interestingly, some experts assigned zero weight to one of the inputs or outputs. This may mean that there is no consensus on the importance of health system inputs and outputs.

The respondents were also asked to estimate the relative weights of three health system outputs: life expectancy at birth, healthy life expectancy, and infant mortality. The respondents considered healthy life expectancy to be the most important output indicator (48.3%). This may be surprising given the fact that life expectancy at birth is the most frequently used indicator. However, the life expectancy at birth is easier to measure than the healthy life expectancy. Experts found life expectancy at birth to be the second most important output (29.9%). Experts consider the infant mortality rate as the least important indicator (21.8%), which is significantly less than the first two health system outputs (Table 2).

The respondents were also asked to choose a country or a group of countries that can serve as benchmarks for the Czech Republic. They could choose one or more of four options: (a) neighboring countries, (b) countries with the most efficient health systems, (c) countries with similar economic development, (d) specify any other country or group of countries. In total, 18 respondents thought that the countries with the most efficient health system should serve as benchmarks for the Czech Republic; five respondents chose countries with the same level of economic development, three respondents chose neighboring countries, and four respondents specified other countries (Austria, Canada, Denmark, Germany, Israel, and the Netherlands) or a group of countries (Southern European countries promoting healthy lifestyle). The finding that two thirds of health policy experts chose countries with the most efficient health systems as the best benchmarks, irrespective of economic development or geographical location, confirms the importance of health system efficiency evaluation among all EU or OECD countries.

**Table 2:** Input and output weights

Indicator	Average weight (%)	Minimum	Maximum
INPUTS			
Health expenditures as % GDP	41.6	12	70
Number of physicians per 1000 inhabitants	22.5	0	39
Number of nurses per 1000 inhabitants	21.4	0	34
Number of beds per 1000 inhabitants	14.5	0	51
OUTPUTS			
Healthy life expectancy at birth	48.3	25	85
Life expectancy at birth	29.9	0	70
Infant mortality rate	21.8	5	50

With the input and output weights available, we can calculate the weighted input and output for each country (Table 3). The lowest weighted inputs were found in Turkey, Poland, and Israel. On the other hand, Korea, Switzerland, and Spain have the highest weighted outputs. The efficiency score was calculated as a ratio of weighted output to weighted input and then normalized to the 0–1 interval by dividing by the maximal value. The application of MCDA (denoted as the MCDA1 model in Table 3) shows that Turkey has the most efficient health system, which is, above all, determined by the very low weighted input. Turkey is clearly an outlier in the given sample of countries. The assumption of the linear relationship between health system inputs and outputs can explain this. Cetin and Bahce [19] also identified Turkey as an outlier compared to other OECD countries. In contrast, Germany, the USA, and Norway have the least efficient health systems.

It should be noted that the weights used in the MCDA models were obtained from experts from the Czech Republic, so they represent local Czech preferences, which may not correspond to the preferences of experts from other countries. At the very least, we can say that we have estimated the efficiency of health systems as seen by Czech experts. This problem does not exist in DEA, where the weights are determined by the DEA model.

The health system of the Czech Republic ranks 16th in the weighted input, 20th in the weighted output and 16th in the health system efficiency. However, we do not recommend that the Czech Republic should follow the countries that are more efficient but achieve lower levels of output. For example, Turkey and Poland are more efficient but achieve worse values in all three health system outputs. The Czech policymakers will hardly accept such a benchmarking recommendation. The Czech Republic should use the countries with higher levels of weighted output with lower levels of weighted input as benchmarks. There are eight such countries: Israel, Estonia, Luxembourg, Italy, the UK, Spain, Slovenia, and Canada (denoted in bold in Table 3). Israel, in particular, can be an excellent benchmark for the Czech Republic because its health system performs very efficiently. Israel uses lower levels of all health system inputs that produce higher life expectancy and HALE. The Czech Republic is better than Israel in infant mortality. In terms of infant mortality rate, the Czech Republic has achieved excellent results.

**Table 3:** Health system efficiency and ranking of countries

Country	Weighted input	Weighted output	Efficiency (MCDA1)	Weighted input ranking	Weighted output ranking	Efficiency ranking
Turkey	0.252	0.948	1.000	1	22	1
Poland	0.396	0.949	0.637	2	21	2
**Israel**	**0.419**	**0.991**	**0.630**	**3**	**4**	**3**
Latvia	0.420	0.924	0.586	4	27	4
**Estonia**	**0.441**	**0.956**	**0.577**	**5**	**19**	**5**
**Luxembourg**	**0.456**	**0.985**	**0.575**	**6**	**10**	**6**
Slovakia	0.459	0.947	0.549	7	23	7
Hungary	0.465	0.933	0.535	8	25	8
**Italy**	**0.503**	**0.990**	**0.524**	**9**	**5**	**9**
**UK**	**0.503**	**0.970**	**0.514**	**10**	**18**	**10**
**Spain**	**0.517**	**0.993**	**0.511**	**12**	**3**	**11**
**Slovenia**	**0.523**	**0.975**	**0.496**	**13**	**17**	**12**
**Canada**	**0.535**	**0.981**	**0.488**	**15**	**12**	**13**
Korea	0.546	0.997	0.486	17	1	14
Russia	0.509	0.902	0.471	11	28	15
Czech Republic	0.545	0.955	0.466	16	20	16
Lithuania	0.533	0.930	0.464	14	26	17
Denmark	0.579	0.977	0.449	18	14	18
Australia	0.585	0.981	0.447	19	11	19
Iceland	0.593	0.990	0.445	20	6	20
Belgium	0.595	0.976	0.437	21	15	21
Sweden	0.610	0.989	0.431	22	7	22
France	0.618	0.989	0.426	23	8	23
Austria	0.691	0.978	0.377	24	13	24
Switzerland	0.732	0.996	0.362	27	2	25
Norway	0.725	0.985	0.362	26	9	26
USA	0.703	0.935	0.354	25	24	27
Germany	0.734	0.976	0.354	28	16	28

In the next step, we investigate the robustness of the original MCDA1 results (Table 4). First, we investigate the effect of a different normalization of the data. In the MCDA2 model, we normalize the data into 0–1 intervals by setting the minimum value to 0 and the maximum value to 1:

5 $$\eqalign{& \>x_{ij}^* = {{{x_{ij}} - mi{n_j}\left( {{x_{ij}}} \right)} \over {ma{x_j}\left( {{x_{ij}}} \right) - mi{n_j}\left( {{x_{ij}}} \right)}},\>i = {\rm{1,2}},{\rm{3,4}}; \cr & \,\,\,\,\,\,\,\,y_{kj}^* = {{{y_{kj}} - mi{n_j}\left( {{y_{kj}}} \right)} \over {ma{x_j}\left( {{y_{kj}}} \right) - mi{n_j}\left( {{y_{kj}}} \right)}},\>k = {\rm{1,2}},3. \cr}$$

Second, we introduce a non-linear relationship between inputs and outputs by using squared values of original health system outputs in the MCDA3:

6 $$\eqalign{& \>x_{ij}^* = {{{x_{ij}}} \over {ma{x_j}\left( {{x_{ij}}} \right)}},\>i = {\rm{1,2}},{\rm{3,4}}; \cr & \,\,\,\,\,\,\,\,\,y_{kj}^* = {{{y_{kj}}^2} \over {ma{x_j}\left( {{y_{kj}}^2} \right)}},\>k = {\rm{1,2}},3. \cr}$$

As a result, in the MCDA3 model, higher levels of outputs are more valued as it is increasingly difficult to improve population health and achieve higher values of outputs.

Third, we used the Healthcare Access and Quality Index (HAQ) as the single health system output in the MCDA4 model. The HAQ is measured on a scale from 0 (worst) to 100 (best) and uses 32 causes of amenable mortality that could be avoided by timely and effective health care. The inputs and outputs are normalized to the 0–1 range as in the MCDA1 model (2).

Spearman rank correlations between each pair of MCDA models are statistically significant at the 0.01 level. In all four MCDA models, Turkey has a leading position in the sample of countries. Israel is considered to be the second most efficient health system. On the other hand, any change in the original MCDA1 model has a very negative effect on the position of Latvia. In all MCDA models, the health system efficiencies of the USA and Germany were very badly evaluated. The position of the Czech Republic in the four MCDA models was relatively stable, with the Czech Republic occupying the 15th to 19th position.

We also calculated the efficiency scores for the output-oriented constant and variable returns to scale (CRS and VRS) DEA models. It is evident that the DEA allowing individual weights for each country has to be less discriminatory than MCDA, so the DEA efficiency scores are higher than the MCDA efficiency scores. The VRS DEA model is usually preferred to the CRS DEA model because the VRS model can express decreasing marginal health system output, which we particularly expect at high output levels. However, the VRS DEA model (Table 4) estimates that all health systems are efficient or nearly efficient. We consider such efficiencies improbable. High values of efficiency scores can result from the curse of dimensionality even though DEA models with four inputs, three inputs, and 28 units fulfil the rule of thumb that the number of units should be equal to or greater than *max*(*m*×*r*, 3(*m* + *r*)), where *m* and *r* are the numbers of inputs and outputs. Spearman rank correlations between each pair of DEA and MCDA models are statistically significant at the 0.05 level, except for (CRS DEA, MCDA1) and (CRS DEA, MCDA3). Both DEA models show low health system efficiencies for the Czech Republic.

**Table 4:** Efficiency evaluation by MCDA and DEA models

Country	MCDA 2	Rank	MCDA 3	Rank	MCDA 4	Rank	DEA CRS	Rank	DEA VRS	Rank
Turkey	1.000	1	1.000	1	1.000	1	1.000	1–4	1.0000	1–15
Poland	0.079	4	0.638	3	0.719	4	0.810	13	1.0000	1–15
Israel	0.109	2	0.659	2	0.773	2	0.945	5	1.0000	1–15
Latvia	0.041	21	0.571	6	0.645	11	0.652	20	1.0000	1–15
Estonia	0.070	7	0.582	5	0.675	7	0.652	19	1.0000	1–15
Luxembourg	0.086	3	0.597	4	0.748	3	0.850	11	1.0000	1–15
Slovakia	0.048	18	0.548	7	0.623	13	0.627	22	0.9970	28
Hungary	0.041	22	0.527	10	0.624	12	0.687	17	0.9990	19
Italy	0.074	5	0.548	8	0.694	5	0.853	10	1.0000	1–15
UK	0.060	10	0.526	11	0.645	10	1.000	1–4	1.0000	1–15
Spain	0.070	8	0.535	9	0.675	6	0.927	7	1.0000	1–15
Slovenia	0.063	9	0.510	13	0.653	9	0.657	18	1.0000	1–15
Canada	0.059	12	0.505	14	0.660	8	1.000	1–4	1.0000	1–15
Korea	0.073	6	0.511	12	0.615	14	0.847	12	1.0000	1–15
Russia	0.010	28	0.450	21	0.517	22	0.778	14	0.9996	16
Czech Republic	0.044	19	0.469	15	0.582	17	0.559	26	0.9989	21
Lithuania	0.029	26	0.456	19	0.496	23	0.624	23	0.9982	25
Denmark	0.050	16	0.463	17	0.575	19	0.897	9	0.9990	20
Australia	0.052	14	0.462	18	0.601	16	0.726	16	0.9983	24
Iceland	0.060	11	0.464	16	0.612	15	0.912	8	1.0000	1–15
Belgium	0.048	17	0.449	22	0.567	20	0.645	21	0.9986	23
Sweden	0.053	13	0.450	20	0.577	18	1.000	1–4	1.0000	1–15
France	0.051	15	0.444	23	0.555	21	0.612	24	0.9981	26
Austria	0.038	24	0.388	24	0.496	24	0.434	28	0.9987	22
Switzerland	0.042	20	0.380	25	0.493	25	0.611	25	1.0000	1–15
Norway	0.040	23	0.376	26	0.486	26	0.736	15	0.9991	18
USA	0.017	27	0.349	28	0.446	28	0.940	6	0.9993	17
Germany	0.035	25	0.364	27	0.461	27	0.460	27	0.9980	27

## Discussion

When evaluating the efficiency of the health system, we encounter many pitfalls that result from the fact that the production of health services differs in many ways from the traditional theory of production as we know it from economic textbooks (e.g. [40, 41]). Below, we review these pitfalls and discuss the approaches we have applied.

*Output measurement.* The Constitution of the World Health Organization defines health as a state of complete physical, mental and social well-being and not merely the absence of disease or infirmity [42]. This definition of health is highly valued due to its comprehensiveness but is also criticized due to the problems in measuring such a broadly defined category. In the empirical research, we have to operationalize the original WHO definition. Hence, a critical methodological problem of health economics is the definition and measurement of the output of the production process: improved health outcomes [43]. Some researchers refrain from measuring health directly and study how health system inputs (physicians, nurses, pharmaceuticals, machines, buildings) produce intermediate outputs such as inpatient days, hospital discharges, consultations, and medical tests. See, for example, survey in [7]. This input-output approach has been widely and successfully used in many industries. Given the problems with health measurement, this is a good approach that researchers can also use in the health sector. Other researchers reject any compromise and use health outcomes as a final output of production in the health sector (e.g. [3, 28]). At least, health outcomes in the form of mortality are usually available (life expectancy, infant mortality). Both approaches to defining health system outputs are possible and can be found in the literature [6, 7, 30]. The view of health outcome as a final output should be preferred in determining the efficient use of health resources. Health care, an intermediate output, may substitute health outcomes in cases where the information on health outcomes is difficult to define and measure. We used final health outcomes in this study.

*Non-homogeneity of production units (health systems).* Dyson et al. [44] systematically studied the pitfalls in the efficiency evaluation by DEA, including problems with non-homogeneity, and suggested a list of protocols to guide researchers. They formulated three assumptions of homogeneity: (1) the production units perform similar activities and produce comparable outputs; (2) the same inputs (resources) are available to all production units; (3) the production units operate in similar external environments. The first two assumptions of homogeneity are related to production units. The third assumption is related to a non-homogeneous external environment under which production units operate. The environment is a set of external factors that affect the efficiency of a production unit but are not usually considered typical inputs in the DEA models and are not under the control of the management. External factors include governmental regulation, social and economic conditions, ownership (public/private), and geographic location. Dlouhý [6] summarized 14 recommendations on how to deal with the non-homogeneity in the health system efficiency evaluation. We applied three of them. First, choose the set of units carefully. We chose the developed countries from the OECD database to include countries with relatively high levels of social and economic development and well-developed health systems. Second, reliable data, ideally from standardized databases, should be used. The non-homogeneity in the data can be a particular problem in studies involving international comparisons in which there is a risk of different national definitions of indicators. The OECD and WHO databases are the best international databases available. Third, be aware of the limited validity of the evaluation. It is still possible to perform an evaluation even if there are doubts about the homogeneity of units. However, the validity of such evaluation has to be subjected to a critical analysis.

*External inputs (health determinants) outside the health system.* The efficiency of the health system is hard to estimate if there are strong effects of several health determinants outside the health system, such as social and economic conditions and physical environment (e.g. [45–47]). According to some empirical research, the effect of health care is only 10–20% [46]. There is a problem with the interpretation of the variables, as it is unclear what they reflect. Female literacy, for example, is associated with a general position of women in the society. In that case, the entire social structure of society is hidden behind that indicator. The role of social determinants on population health is complex and poorly understood. In extreme cases, any decision by the government or an individual has a direct or indirect impact on health. However, we are unable to investigate and analyze such complex relationships. As a solution, Nolte and McKee [48] suggest using the concept that deaths from certain causes should not occur in the presence of timely and effective health care. Such mortality indicators are known as avoidable mortality and mortality amenable to health care. Nolte and McKee [48] found that rankings based on mortality amenable to health care differed substantially from rankings of health attainment in the *World Health Report 2000*. Dlouhý [6] compared DEA models with life expectancy at birth and with the *Healthcare Access and Quality Index* (HAQ) as the output variable. The HAQ is measured on a scale from 0 (worst) to 100 (best) and uses 32 causes of amenable mortality that could be avoided by timely and effective health care [28, 49]. Except for one country (Lithuania), the DEA models give similar results. We used the HAQ index as a single health system output in the MCDA4 model.

*No free lunch.* The basic assumption of the production theory is that there are no outputs without inputs. However, even with no health system, people will live, and their life expectancy will not reach zero. So, there indeed are positive health system outputs. A solution could be to determine the value of health indicators independent of the existence of a health system and subtract these values from the actual values of the indicators. The MCDA1 and MCDA2 models we used represent two extremes of this approach because MCDA1 considers zero values of health indicators as a basis, and MCDA2 considers the minimum real value of each health indicator as a basis.

*Isotonicity property (output maximization and input minimization).* In production theory, inputs are minimized, and outputs maximized. The isotonicity property requires that an increase in any input should not result in a decrease in any output [44, 50]. Thus, the values of some inputs or outputs may have to be transformed. We will maximize output as life expectancy but not the infant mortality rate, which we need to transform into the infant survival rate in this study. The isotonicity property is not a critical problem but rather a technical issue known from MCDA and DEA applications, and it is not restricted to health care.

*Inter-temporal input-output dependence*. The static efficiency models fail in the presence of inter-temporal input-output dependencies because a causal correspondence between coincident inputs and outputs, which is a fundamental assumption of a production theory, is not met. For example, such inter-temporal dependences may arise when capital stock is used as an input because the capital stock affects output levels over many subsequent periods. A typical healthcare example is prevention with long-term health effects. Emrouznejad and Thanassoulis [51] developed a dynamic DEA model in which inter-temporal dependence is modeled by paths of coincident input and output levels. For example, depreciation is a way to measure the value of the capital stock in monetary terms. Using average values of health system inputs and outputs for more years can be a partial solution if panel data are available. To the best of our knowledge, this issue has not been addressed in health system efficiency evaluations. The reason is apparently that the potential impacts are considered negligible.

*Selection of benchmarks*. DEA and SFA clearly define benchmarks (peer units) for inefficient units. In MCDA, it is less clear whether a benchmark should be the unit ranked first. However, some benchmarks can be seen as unacceptable by policymakers. As discussed above, in MCDA1, Turkey and Poland are more efficient than the Czech Republic, but they achieve worse values for all three health system outputs. Such a recommendation will hardly be accepted by policymakers. We suggest using benchmark countries that dominate the Czech Republic in terms of both weighted output and weighted input.

## Conclusion

Policymakers, who are constantly discussing growing health expenditures, should know whether health care is provided efficiently. Researchers can provide such information through health system efficiency evaluations. In this paper, we used MCDA and DEA to evaluate the efficiency of the health systems of 28 developed countries. The models included four health system inputs and three health system outputs. According to the MCDA models, Turkey, Poland, and Israel were found to have efficient health systems. The Czech Republic ranked 16th, 19th, 15th, and 17th in the MCDA models. The benchmark countries for the Czech Republic’s health system identified by the MCDA1 model were Israel, Estonia, Luxembourg, Italy, the UK, Spain, Slovenia, and Canada (Table 3). These countries use lower weighted health system inputs while achieving better health outcomes than the Czech Republic. The output-oriented CRS DEA model identified four efficient health systems: Turkey, the UK, Canada, and Sweden. The Czech Republic was found to be one of the worst-performing health systems. The output-oriented VRS DEA model did not provide useful results, as 15 countries were efficient. Nevertheless, the Czech Republic was inefficient again.

The efficiency of the Czech health system can be achieved by reducing health system inputs and improving health system outputs. However, it is politically difficult to reduce the number of doctors and the number of nurses or decrease health expenditure. It is acceptable to reduce the number of beds by 10% through efficiency improvement measures. On the output side, we cannot expect an improvement in the infant mortality rate, where the Czech Republic is already achieving excellent results. Extension of life expectancy by 1.5 years and healthy life expectancy by one year can be achieved through efficient use of health system inputs and effective public health policies. For example, in the CRS DEA model, the efficiency score will increase from 0.559 to 0.570.

The contribution of this paper to the literature is threefold. First, we obtained subjective information on the relative importance of the health system inputs and outputs from Czech health policy experts. DEA and SFA do not provide such information because the importance of inputs and outputs is determined “objectively” by the model. Second, we were able to evaluate the health system efficiency by MCDA and DEA in one study. Two review studies on the health system efficiency [30, 31] did not mention any application of MCDA. Third, during the model formulation, we investigated the pitfalls of efficiency measurement in health care and used several practical solutions.

This study has several limitations. First, we must deal with data availability because some countries had to be omitted due to missing data. Second, the comparability of data is a traditional problem of international comparisons, even if we use the OECD data. Third, there needs to be a higher number of health system input and output indicators in the models. This can be realized in the MCDA evaluation, but not in DEA evaluation, which is sensitive to the curse of dimensionality. Fourth, outliers can shift a production frontier extremely. We have shown that in the case of Turkey. Fifth, the production of population health is a multifactorial and complex issue, so it is practically impossible to make the right choice of crucial health system inputs. Sixth, no single performance evaluation framework or method provides a universal guide to determining whether a health system is efficient compared to other health systems. It is appropriate to combine different methods. We consider MCDA and DEA as exploratory methods, not methods providing definitive answers [21, 31]. Seventh, the health system is a relatively large and complex system, so the practical interpretation of the results is looser in contrast to strict theoretical assumptions. This is consistent with the view that MCDA and DEA are exploratory methods.

In future research, we will consider three important challenging topics. First, we should continue with the comparison of various efficiency evaluation methods. Most efficiency studies use DEA to assess health system efficiency, while applications of MCDA are less frequent. It is a good recommendation for further research to compare these two methods and determine if the results systematically differ if the input and output weights are determined by experts (MCDA) or the method itself (DEA). Second, we should concentrate the research on the impact of the COVID-19 pandemic, which raises several research questions. One way to achieve efficiency in the health system is by lowering the number of health system capacities. However, lower health system capacities can be overwhelmed during a sudden outbreak, as observed during the COVID-19 pandemic. Consequently, the aim of efficiency must be redefined because some reserve capacity that policymakers considered inefficient in the past is needed to ensure system resilience. Health systems must function more flexibly, and health system inputs must be mobilized quickly in case of unexpected demand. Third, we need to develop evaluation models that are able to deal with the fact that the production of health is quite a complex process. The health system efficiency is not only a function of health system inputs and outputs describing the production technology but is also affected by external factors such as the lifestyle, environmental and socio-economic conditions. Estimating and separating their impact on health system efficiency is a major research challenge.

## Data Availability

The OECD data used in this study (Table 1) are publicly available at 10.1787/health-data-en. The healthy life expectancy (HALE) data are publicly available from the WHO database at https://www.who.int/data/gho/data/indicators/indicator-details/GHO/gho-ghe-hale-healthy-life-expectancy-at-birth. The HAQ data are available from publication [49].

## Abbreviations

CRS: Constant Returns to Scale
DEA: Data Envelopment Analysis
EU: European Union
FDH: Free Disposable Hull
GDP: Gross Domestic Product
HAQ: Healthcare Access and Quality Index
MCDA: Multiple-Criteria Decision Analysis
OECD: The Organization for Economic Co-operation and Development
PPP: Purchasing Power Parity
SFA: Stochastic Frontier Analysis
VRS: Variable Returns to Scale
WHO: World Health Organization
